# Patterns of biodiversity response along a gradient of forest use in Eastern Amazonia, Brazil

**DOI:** 10.7717/peerj.8486

**Published:** 2020-02-11

**Authors:** Sérgio G. Milheiras, Marcelino Guedes, Fernando Augusto Barbosa Silva, Perseu Aparício, Georgina M. Mace

**Affiliations:** 1 Centre for Biodiversity and Environment Research, University College London, London, United Kingdom; 2 School of Natural and Environmental Sciences, Newcastle University, Newcastle-upon-Tyne, United Kingdom; 3 Embrapa-Amapá, Macapá, Amapá, Brazil; 4 Instituto de Ciências Biológicas, Universidade Federal do Pará, Belém, Pará, Brazil; 5 Universidade Estadual do Amapá, Macapá, Amapá, Brazil

**Keywords:** Amazon forest, Brazil nuts, Amapá, Pará, Forest management, Nymphalidae, Scarabaeinae, Selective logging

## Abstract

The preservation of tropical forests is increasingly at risk, including forests located within human-modified landscapes that retain high conservation value. People modify and interact with these landscapes through a wide range of uses. However, our knowledge of how different forest uses affect biodiversity is limited. Here, we analyse the responses of different taxa to four distinct categories of forest management, namely old-growth forest, Brazil nut extraction areas, reduced impact logging areas, and eucalyptus plantations. Within six independent replicates of each category, we sampled three taxa (fruit-feeding butterflies, dung beetles, and trees) in eastern Amazonia. Forests under moderate use (Brazil nut extraction and reduced-impact logging) had similar, albeit slightly lower, diversity levels relative to old-growth forests, while communities in plantations were significantly less diverse. Only 4%, 20%, and 17%, of the sampled butterfly, dung beetle, and tree species, respectively, were restricted to old-growth forests. This study provides further empirical evidence of the importance of old-growth forest conservation in the context of human-modified landscapes. It also suggests that landscape matrices integrating forest uses at varying intensities are well positioned to reconcile biodiversity conservation with the production of goods that support local livelihoods.

## Introduction

Tropical human-modified landscapes are characterised by vegetation patches under varying levels of use intensity and habitat degradation, which influence their capacity to retain biodiversity and provide ecosystem services (Melo et al., 2013). The presence of undisturbed natural habitats in these landscapes has clear benefits for local biodiversity (Gibson et al., 2011). But in systems where undisturbed areas are scarce, forests under some degree of anthropogenic disturbance can help buffer the impact of more intensely managed land (Bhagwat et al., 2008;  Santos-Heredia et al., 2018). Furthermore, the benefits extracted from these forests might be crucial for achieving sustainable landscape configurations (McNeely & Schroth, 2006; Nobre et al., 2016). Yet, we still have limited knowledge of how different components of human-modified landscapes, including their animal and plant communities, are affected by the full spectrum of use intensities that are common in many tropical regions (Chazdon et al., 2009).

Forest use intensification tends to lead to diminished biodiversity (Barlow et al., 2007a; Davis, Huijbregts & Krikken, 2000; Edwards et al., 2014; Gardner et al., 2008; Nichols et al., 2007; Wilcove et al., 2013). Selectively logged forests, for example, can retain most of the species found in old-growth forest (Ghazoul, 2002; Hamer et al., 2003; Lewis, 2001; Ribeiro & Freitas, 2012; Slade, Mann & Lewis, 2011), but that is dependent on the volumes harvested or the duration of rotation cycles (Burivalova, Şekercioğlu & Koh, 2014; Edwards et al., 2014; Richardson & Peres, 2016). Furthermore, ecological processes may be negatively affected even under low logging intensities (França et al., 2017).

The comparative biodiversity studies that assess these impacts benefit from multiple taxa approaches due to possible idiosyncratic responses from specific taxonomic groups (Kessler et al., 2009; Beiroz et al. 2017). Invertebrates respond rapidly to disturbance due to their short generation times and high population growth rates (Sodhi et al., 2010). Both fruit-feeding butterflies and dung beetles have additional characteristics that increase their suitability as ecological indicators, such as relatively large body sizes, ease of sampling and a relatively well-known taxonomy (Ribeiro & Freitas, 2012). In both taxa the species with restricted geographic ranges or forest specialists tend to be particularly vulnerable to disturbance (Cajaiba et al., 2017; Davis, Huijbregts & Krikken, 2000; Lewis, 2001; Sodhi et al., 2010). But their responses to disturbance are not necessarily congruent due to different life histories (Davis, Huijbregts & Krikken, 2000; Schulze et al., 2004). The feeding specialisations seen in some butterfly species are less common in dung beetles, for example. On the other hand, the involvement of dung beetles in ecosystem processes is better studied, including their impact on nutrient cycling, bioturbation, and secondary seed dispersal (Nichols et al., 2008;  Santos-Heredia et al., 2018).

Trees are the main structural components of forests and tree community change might in fact help predict changes in other taxa (Barlow et al., 2007a; Bobo et al., 2006). Trees are severely affected by land use intensification (Gerstner et al., 2014; Philpott et al., 2008; Schulze et al., 2004). Tree communities might not resemble those of intact forests even several decades after disturbance (Richardson & Peres, 2016; Sodhi et al., 2010). Furthermore, changes in species composition can take years to manifest, including seedling regeneration (Darrigo, Venticinque & Dos Santos, 2016). While the Amazonian tree flora is one of the most diverse globally, its species and associated extinction risk are in many cases poorly known (Ter Steege et al., 2015). Tree species with certain traits, such as reliance on mammal pollinators, might be particularly vulnerable to disturbance (Sodhi et al., 2010).

Here, we examine the spatial distribution of fruit-feeding butterfly, dung beetle, and tree communities in a landscape with varying forest management regimes. Our main objective is to compare the conservation value, i.e., the capacity to support biodiversity, of four relevant forest uses in northeast Amazonia, namely old-growth forest, Brazil nut extraction areas, selective logging areas, and eucalyptus plantations. While old-growth forest is sampled here as baseline, the focus of this research is on forests directly used by people, therefore excluding secondary forests, which mostly benefit people indirectly, as a vital component of the shifting agriculture cycle (Brown & Lugo, 1990). The different research questions that drive this study are: can moderate and intensive forest uses maintain the same levels of richness, abundance and diversity found in old-growth forests? Do different taxa exhibit the same responses to forest use intensification? And can moderate and intensive use forests have a role in sustainable human-modified landscapes? Here we: (i) compare the levels of species richness, abundance and diversity of the three sampled taxa between old-growth forests, Brazil nut extraction areas, selective logging areas, and eucalyptus plantations; (ii) assess if fruit-feeding butterflies, dung beetles, and trees exhibit congruent responses to forest use change; (iii) explore the implications this research to nature conservation and forest management in the tropics.

## Methods

### Study site

**Figure 1:**
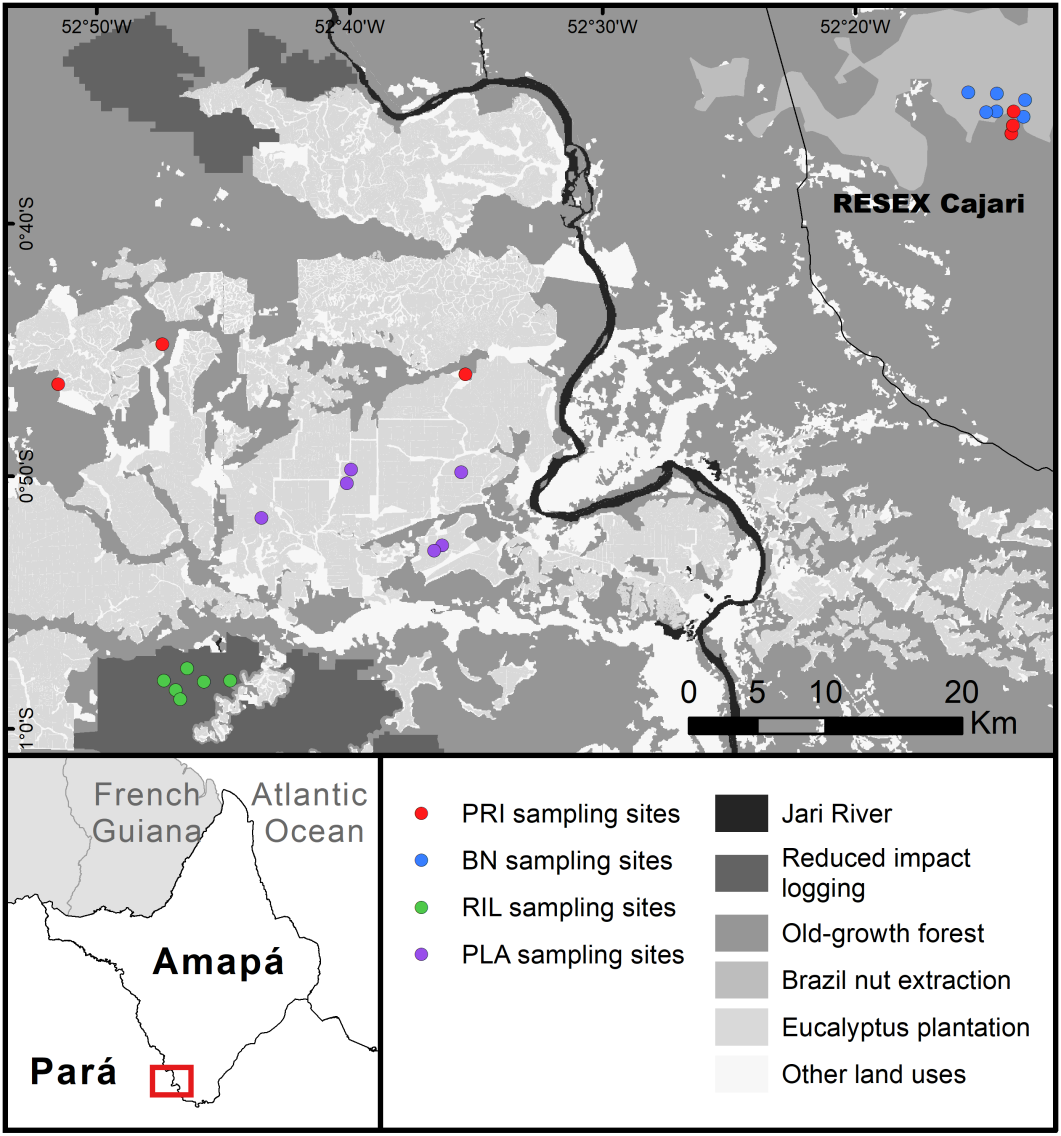
Map of the study area. Abbreviations refer to old-growth forest (PRI), Brazil nut extraction (BN), reduced impact logging (RIL), eucalyptus plantation (PLA). RESEX is a protected area category in Brazil. BN delineates the area where Brazil nut trees have been mapped within the RESEX (Costa, 2018). Latitudinal and longitudinal coordinates are indicated at the margins of the map.

The study was undertaken at the east–west border between Amapá and Pará (1°0′S–0°30′S; 53°0′W–52°10′W), two states in the Brazilian Amazon forest (Fig. 1). Forest cover is relatively high, while human density is low and concentrated on the margins of Jari River. The landscape is characterised by a large eucalyptus plantation and natural forest under different management regimes, with relatively little agriculture. The climate is tropical, the mean annual rainfall is around 2,300 mm, with a wetter season from January to June, and mean temperature around 27 °C throughout the year (INMET, 2018). Soils are predominantly ferralsols and acrisols (IBGE, 2003). Sampling points ranged between 45–217 metres above sea level, with slopes of 0.1–9.2 degrees.

The sampled eucalyptus (*Eucalyptus urograndis*) plantations lie mostly on the Pará side of the study area. The sampled areas were planted between 2011 and 2013. The sampled selective logging areas were cut down, for the first time, in 2013 and 2014, with the timber extracted ranging from 9.5–16.2 m^3^ ha^−1^ (3–7 trees ha^−1^). Brazil nut trees (*Bertholletia excelsa* Bonp.) tend to have an uneven distribution throughout their range, occurring in clusters (Mori & Prance, 1990), locally called *“castanhais”*. Within the study area, *“castanhais”* are mainly located in the sustainable use reserve RESEX Cajari (IUCN protected area category VI), where sampling was carried out. Brazil nut extraction is legally protected within the RESEX and is managed by the local community, while commercial logging is illegal. Brazil nut trees used locally were mapped as part of a project in collaboration with the state government (Costa, 2018). Local fruit fall and collection occur mostly between January and April. Old-growth forest sampling sites were located in *terra firme* (non-flooded) dense forest, half in the western part of the study area, the other half inside the RESEX in the eastern part of the study area. These sites reflect the conditions of old-growth forest within a landscape with human presence. They are relatively accessible and therefore likely to experience low to moderate levels of human disturbance (e.g., hunting). The distance to the closest secondary road in old-growth forest sampling points ranged from 1.2 to 4.2 km.

We sampled a total of 24 sites, six sites in each forest use category. Sampling was conducted at the same locations for dung beetles, butterflies, and trees (Fig. 1). Butterfly and dung beetle traps were installed at least 30 metres from the tree plots, in opposite directions (i.e., at least 100 metres distance between butterfly and dung beetle traps). All sampling sites were separated by more than 500 m (range: 0.6–76.1 km; mean: 37.3 ± 27.0 km). Site location was also constrained by accessibility and a minimum distance to edge of 100 m. We used a Garmin GPSMAP 64s for georeferencing. Fieldwork was authorised by Instituto Chico Mendes de Conservação da Biodiversidade (process number: 56120-1) and Conselho Nacional de Desenvolvimento Científico e Tecnológico (process number: 01300.001010/2016-16).

### Dung beetle sampling

Dung beetles (Coleoptera: Scarabaeinae) were sampled, using baited pitfall traps, in May-July 2017 with replication in October-December 2017, covering the peaks of the wet and dry seasons to account for seasonality (Korasaki et al., 2013). Each sampling unit consisted of three traps placed three meters apart in a triangular arrangement. Traps were collected after 24 h. Previous studies have shown that 24 h sampling periods for dung beetles produce reliable results (França et al., 2016). Trap configuration consisted of a rain cover (plastic plate with 25.5 cm diameter, placed 16 cm from the ground), below which a meshed nylon bag containing the bait was suspended, directly above a buried plastic container (13 cm height × 11.5 cm diameter). A third of the container was filled with salted water. The bait used was human faeces (∼30 g), which is an effective bait for dung beetles (Marsh et al., 2013). Specimens were stored in alcohol at 70%. After initial triage by morphospecies, the material was sent to the Federal University of Pará for species identification by one of the co-authors (F.A.B.S.).

### Butterfly sampling

Frugivorous butterflies (Lepidoptera: Nymphalidae) were sampled using fruit-baited cylindrical traps (van Someren-Rydon traps), following established guidelines (Lucci Freitas et al., 2014; Van Swaay et al., 2015). Fieldwork took place between May–July 2017 and was replicated between October–December 2017 to account for seasonal fluctuations (Hamer et al., 2005a; Hamer et al., 2005b). Each sampling unit consisted of a linear transect with four traps separated by 30 m. The base of the traps hung between 1 m and 1.5 m above the ground. The bait was banana fermented for 48 h. After installation, traps remained in the field for six days and were visited every 48 h to replace the bait and record the individuals captured. At least one voucher specimen per species was retained as reference. The remaining captured individuals were identified, photographed with a macro lens, marked with a black or silver marker and released. The collected individuals were sent to State University of Campinas in São Paulo, where their identification was validated by the team of Prof. André Lucci Freitas. Recaptures were not used in the analyses to avoid overestimating butterfly abundance (Ribeiro et al., 2008). The main identification references used were Warren et al. (2013), Neild (1996), Neild (2008), and D’Abrera (1987), D’Abrera (1988).

### Tree sampling

Trees with diameter at breast height (DBH) equal or higher than 10 cm were sampled in 0.4 ha plots (100 × 40 m). Sampling was conducted between July and October 2017. Plot establishment followed the guidelines from Phillips et al. (2016). Six plots were set in each forest use under analysis, totalling 24 plots. The plots were first stringed, and then coordinates, elevation and slope were registered at each plot corner and its centre. Plot orientation was equally divided between N-S and E-W. All sampled trees were tagged. Voucher photos were taken of each species, with a small cut in the trunk and leaf close-up when available. When buttresses prevented the measurement of DBH, it was calculated using digital camera photos, as described in Phillips et al. (2016). Species identification was carried out by three experienced local parataxonomists. Common names were converted to scientific names using the species list developed for the local tree community by the selective logging company operating locally and were checked for typos using ‘BIOMASS’ R package.

### Environmental variables

We measured slope, elevation, and sand percentage in the soil at each sampling point to control for environmental heterogeneity. Soil samples were analysed at Embrapa laboratories in Macapá, Amapá. For each site the combined sample was collected with a soil auger for the 0–10 cm layer and consisted of a soil mixture from five subplots separated 50 m along a linear transect. Slope and elevation were measured at each site with a Haglöf EC-II electronic clinometer and a Garmin GPSMAP 64s, respectively. Slope was determined by measuring the angle, at 10 metres distance, of a reference point at eye height. Plot level slope and elevation are an average of seven measurements taken at the centre and borders of the tree plots.

### Data analysis

We measure species diversity with the Simpson diversity (using the formula $1/{\mathop{\sum }\nolimits }_{i=1}^{s}{p}_{i}^{2}$, where *p*_*i*_ is the proportional abundance of species and *s* the number of species) (Jost, 2006). Since observed species richness is likely to be underestimated (Brose, Martinez & Williams, 2003), we also calculated the species richness estimator JACK2 to estimate the species pool in each sampled forest type. Kruskal-Wallis rank sum chi-squared tests were conducted to test the homogeneity of observed species abundance distributions between forest uses (Table S1) and to compare the sampled insect communities between both seasons.

To analyse the differences in species richness, abundance and diversity between the sampled forest uses we used generalised linear models with a Gaussian error distribution. Old-growth forest is the reference level in the forest use categorical variable. The models assess the relationship between richness, abundance or diversity (response variables) and forest use (explanatory variable) and are also adjusted for three environmental variables: slope, elevation, and sand percentage in the soil. Sand percentage is used as indicator of soil texture, due to its high correlation with silt and clay (Gries et al., 2012). By including these three environmental variables in our models we aim to understand if natural factors are driving the variation in our response variables. We use *t*-tests to test the significance of the model coefficients. We checked the residuals in all models to evaluate the adequacy of the error distribution. Furthermore, we conducted non-metric multidimensional scaling (NMDS), using the Bray–Curtis dissimilarity index, to represent the patterns of assemblage composition in the insect taxa sampled. The ANOSIM (analysis of similarities) R statistic was also calculated to assess if there were significant composition differences between all forest uses. Tree NMDS was not carried out due to the artificial tree composition in plantations. Finally, we analysed the share of species found in moderate/intensive uses that also occurred in old-growth forest and the share of species unique to each forest use (Barlow et al., 2007a). In the latter, we divided the number of species occurring solely in one forest use by the total observed richness for the respective taxon. To determine if fruit-feeding butterflies, dung beetles, and trees exhibit congruent responses to forest use change, we used the Spearman’s rho coefficient, a rank-based measure of association, to assess the correlation of the richness, abundance and diversity metrics between the sampled taxa.

Unless stated otherwise, all analysis was conducted in R3.4.3. R Core Team (2017) in packages ‘vegan’ and ‘stats’. All plots were created using ‘ggplot2’. The map in Fig. 1 was developed in ArcGIS 10.3. We use the following abbreviations for each forest use: PRI- old-growth forest; BN- Brazil nut extraction; RIL- reduced impact logging; and PLA- eucalyptus plantations.

## Results

**Figure 2:**
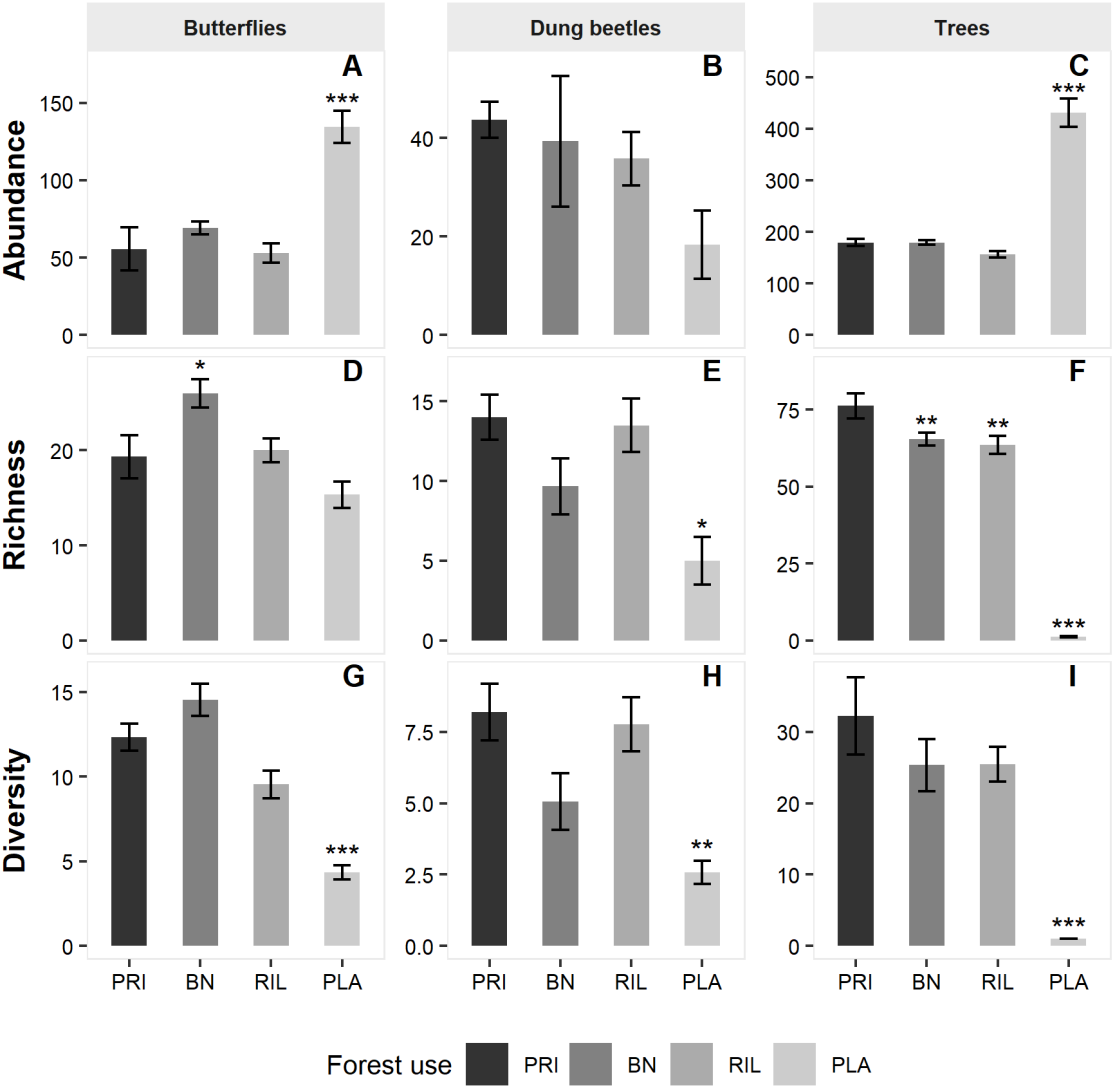
Mean abundance (A–C), richness (D–F) and diversity (G–I) levels across sampling sites per forest use. Error bars represent standard error of the mean. Asterisks indicate significant differences (*** *p*-value ≤ 0.001; ** *p*-value ≤ 0.01; * *p*-value ≤ 0.05) relative to old-growth forest (PRI). BN, Brazil nut extraction; RIL, reduced impact logging; PLA, eucalyptus plantation. Full modelling results are available in Table S2.

We sampled a total of 1,872 butterflies of 78 different species (Table 1), from subfamilies Charaxinae, Biblidinae, Nymphalinae and Satyrinae. Butterfly species richness was 42 in old-growth forest (PRI). It increased to 50 in Brazil nut extraction areas (BN) and to 44 in the reduced impact logging (RIL) areas but decreased to 37 in the eucalyptus plantations (PLA). BN had significantly higher butterfly richness relative to old-growth forest (*β* = 4.69, *p*-value = 0.048; Fig. 2 and Table S2).

For dung beetles, we sampled 823 individuals of 59 different species (Table 1), from tribes Ateuchini, Delthochilini, Coprini, Oniticellini, Onthophagini and Phanaeini. Dung beetle species richness was 38 in old-growth forest. It decreased to 24 species in BN and 29 in RIL and was the lowest in PLA with 19 species. Richness was significantly lower in PLA relative to old-growth forest (*β*= −6.20, *p*-value = 0.044; Fig. 2 and Table S2).

We sampled 5,674 trees belonging to 287 different species and 48 different families (Table 1). Old-growth forest sites had the highest number of species (216), which decreased to 156 in BN sites and 163 in RIL sites. As expected, eucalyptus plantation sites had negligible levels of tree species richness. All forest uses had significantly lower tree richness levels relative to old-growth forests (BN: *β* =  − 11.90, *p*-value = 0.006; RIL: *β*= −15.66, *p*-value = 0.004; PLA: *β* = −70.15, *p*-value < 0.001; Fig. 2 and Table S2).

Model results on abundance (Fig. 2 and Table S2) show that both butterfly (*β* = 69.72, *p*-value = 0.001) and tree (*β* = 231.09, *p*-value < 0.001) abundance were significantly higher in eucalyptus plantations than in old-growth forest. Finally, the diversity metric was significantly lower in PLA relative to PRI for all the sampled taxa (butterflies: *β* =  − 8.53, *p*-value < 0.001; dung beetles: *β* =  − 4.14, 0.008; trees: *β* =  − 28.84, *p*-value < 0.0010).

**Table 1:** Observed species richness (S_obs), estimated species richness (S_est), abundance (N), and Simpson diversity (1/D), per taxon and forest use.

	Butterflies				Dung beetles				Trees			
	*S_obs*	*S_est*	*N*	*1/D*	*S_obs*	*S_est*	*N*	*1/D*	*S_obs*	*S_est*	*N*	*1/D*
PRI	42	62	334	20.09	38	51	262	15.99	216	324	1076	73.00
BN	50	62	415	19.22	24	37	236	6.47	156	231	1076	39.13
RIL	44	61	317	11.49	29	43	215	11.00	163	246	938	39.15
PLA	37	61	806	5.69	19	32	110	6.71	2	4	2584	1.00
Total	78	100	1872	18.66	59	86	823	19.43	287	409	5674	4.74

**Notes.**

PRI old-growth forest BN Brazil nut extraction RIL reduced impact logging PLA eucalyptus plantation

**Figure 3:**
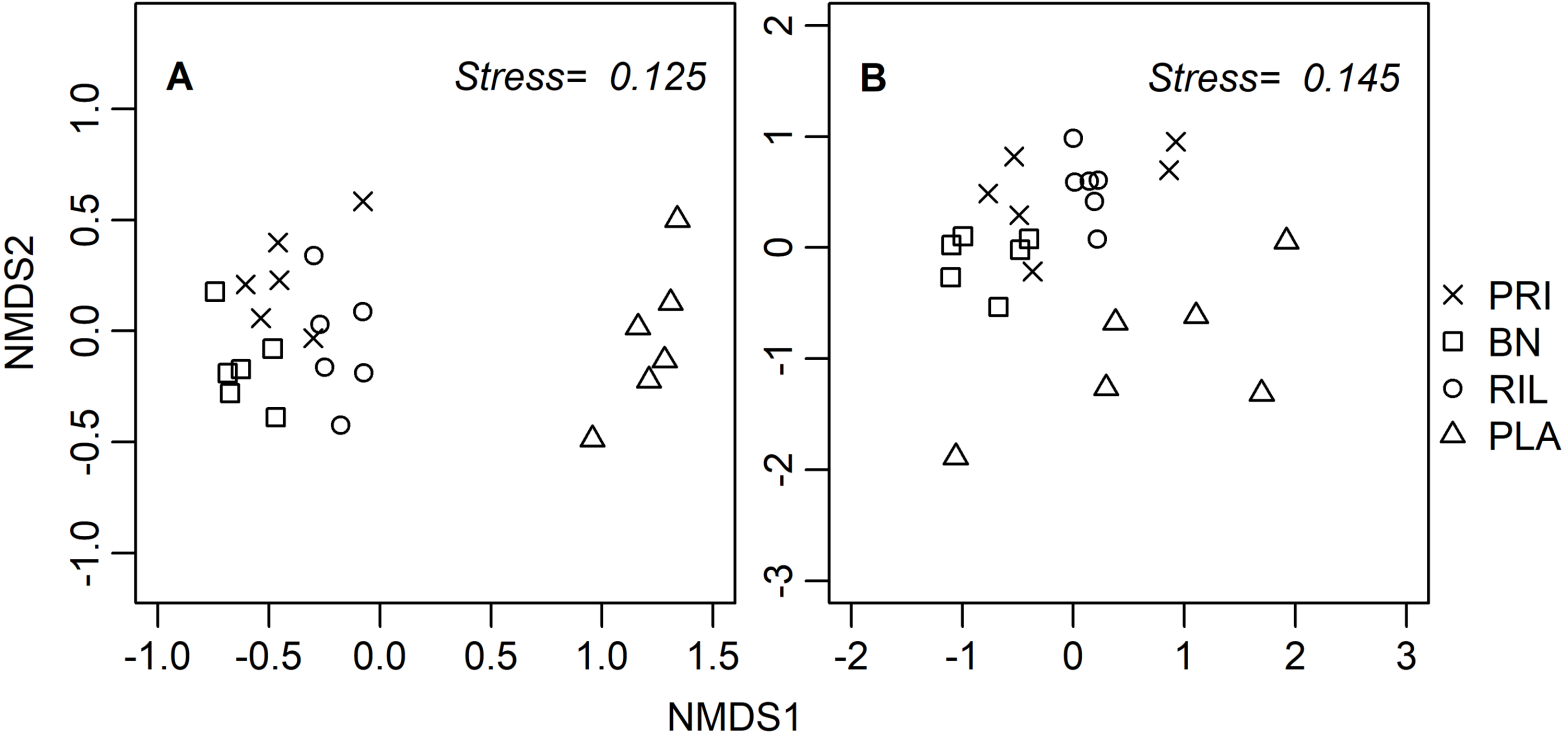
NMDS site ordination diagrams for (A) butterflies and (B) dung beetles. Each point represents one of the 24 sampling sites. Trees were not included due to the tree species composition in eucalyptus plantations. PRI, old-growth forest; BN, Brazil nut extraction; RIL, reduced impact logging; PLA, eucalyptus plantation.

Ordination diagrams, obtained through a distance-based method (NMDS), for dung beetles and butterflies (Fig. 3) provide visual representation of the similarity of communities in BN, old-growth, and RIL forests, while eucalyptus plantations form an independent cluster. The ANOSIM R statistic for butterflies (*R* = 0.78, *p*-value = 0.001) and dung beetles (*R* = 0.53, *p*-value = 0.001) confirms that communities were more similar within the same forest use relative to other uses.

**Figure 4:**
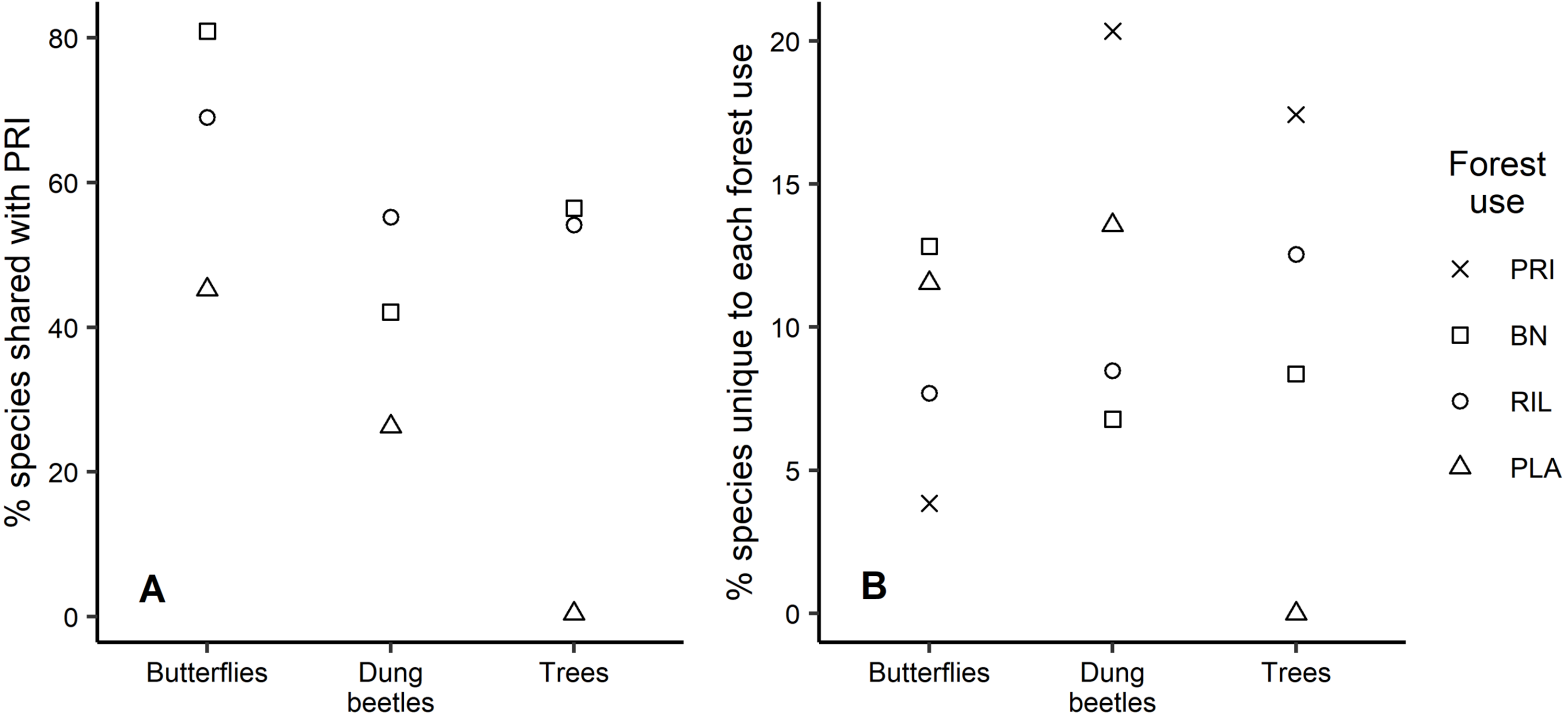
Species uniqueness between forest uses. (A) Percentage of species shared between old-growth forest and the other forest uses, per taxon; (B) Percentage of species unique to each forest use relative to observed richness, per taxon.

Analysis of the species that are shared between old-growth forest and other forest uses shows similar patterns across taxa, with plantations having the lowest percentage of species also occurring in old-growth forest (Fig. 4A). For example, 81% of the butterfly species occurring in old-growth forest were also found in BN sites, which was the highest percentage observed. We found that old-growth forest had the highest share (20.3%) of unique dung beetle species, in contrast with the response observed for butterflies, where it had the lowest share (3.8%) relative to other forest uses (Fig. 4B). Plantations registered the second largest share of unique species for both insect taxa (Fig. 4B). Although eucalyptus trees only occurred in plantations, they have not been considered in Fig. 4B due to their exotic status.

Cross-taxa congruence between dung beetle and butterfly richness or abundance was low (Table 2). There was however a positive correlation between both dung beetle and butterfly richness and tree richness. The same result was observed for the diversity metric. The higher abundance of both butterflies and trees in eucalyptus plantations contributes to the positive correlation between them, while the relationship is reversed between tree and dung beetle abundance. The impact of plantations in these results was confirmed by excluding plantation data from the analyses, which resulted in no significant cross-taxa associations for neither of the biodiversity metrics analysed.

A comparison of the dung beetles and butterflies sampled, across all sites, between the wetter and drier seasons showed distinct seasonal trends, with butterflies having significantly higher levels of abundance (*χ*^2^ = 14.494, *p*-value < 0.001) and richness (*χ*^2^ = 5.820, *p*-value = 0.016) in the wetter season, but not diversity (*χ*^2^ = 0.287, *p*-value = 0.592). For dung beetles, the differences between seasons were not significant for any metric analysed. Sites with higher butterfly richness (rho = 0.590, *p*-value = 0.002), abundance (rho = 0.454, *p*-value = 0.026) and diversity (rho = 0.472, *p*-value = 0.021) were roughly the same in both seasons, while for dung beetles that correlation was only found for diversity (rho = 0.545, *p*-value = 0.006). Plantations accounted for most of the increase in butterfly abundance in the wetter season.

**Table 2:** Pairwise correlation across sites, for species richness, abundance and diversity, between the different taxa analysed. Correlation coefficients were calculated using Spearman rho.

	Dung beetles	Butterflies
*Richness*		
Butterflies	0.064	
Trees	0.503^*^	0.414^*^
*Abundance*		
Butterflies	−0.320	
Trees	−0.412^*^	0.609^**^
*Diversity*		
Butterflies	0.305	
Trees	0.445^*^	0.712^***^

**Notes.**

*** *p*-value ≤ 0.001.

** *p*-value ≤ 0.01.

* *p*-value ≤ 0.05.

## Discussion

### Response to forest use change

The forest management regimes sampled in this study support differing levels of species richness, abundance and diversity of dung beetles, fruit-feeding butterflies and trees. Overall, our results indicate that forest use intensity reduces biodiversity in tropical forest landscapes (Barlow et al., 2016; Gibson et al., 2011), selectively logged forests retain considerable conservation value (Berry et al., 2010; Edwards et al., 2014) and eucalyptus plantations are not necessarily devoid of biodiversity (Barlow et al., 2007a; Gardner et al., 2008). Additionally, our results show that forests used for Brazil nut extraction retain richness levels similar to reduced impact logging, in contrast to what was found by Gibson et al. (2011), where impacts of the category “other extracted forests” are closer to agroforestry or plantations.

The effects on richness found for dung beetles are relatively strong when compared with other studies (Berry et al., 2010; Gardner et al., 2008; Nichols et al., 2007). For example, while the review by Nichols et al. (2007) found a mean change in total richness relative to old-growth forest of −10% for reduced-impact logging and −40% for plantations, those values were −23.7% and −50.0%, respectively, in this study. Tree richness showed a similar pattern to dung beetles, although with the inevitably drastic reduction in eucalyptus plantations. Interestingly, we found similar tree richness decreases in Brazil nut extraction areas (−27.8%) and reduced-impact logging (−24.5%), which might be a consequence of the authorised non-commercial logging carried out by the RESEX community. Brazil nut areas in the Amazon might also be associated with higher historical anthropogenic influence (Levis et al., 2017). On the other hand, butterflies were the exception to the general trend, with richness numbers in moderate uses slightly higher than in old-growth forests (BN: +19.0%; RIL: +4.8%) and only slightly lower under intensive use (PLA: −11.9%). Similar response patterns have been previously observed and are indicative of butterfly communities with higher co-occurrence of species specialised to distinct habitats as a result of disturbance (Fermon et al., 2005; Sant’Anna et al., 2014a; Sant’Anna et al., 2014b).

For conservation, it is relevant to understand how many species present in low disturbance areas are retained in other forest uses, as these species tend to be more specialised and vulnerable to habitat alteration (Fermon et al., 2005; Sodhi et al., 2010). When considering the percentage of species that are unique to each forest use, values were generally low. Barlow et al. (2007a) found ca. 22%, 32%, and 57%, of butterfly, dung beetle, and tree species, respectively, occurring only on old-growth forests, while here those values were 4%, 20%, and 17%. These lower values reflect the closer similarity between communities in old-growth forests and the moderate use forests studied here and absent in Barlow et al. (2007a). Analysing species uniqueness from a different perspective, Barlow et al. (2007a) found that ca. 60% and 42% of butterfly and dung beetle species occurring in old-growth forests were shared with plantations, while results here indicated 45% and 26%, respectively. Other studies have also reported relatively higher levels of shared species than those found here (Berry et al., 2010; Edwards et al., 2014; Nichols et al., 2007). The relatively high average distance between sampling points in this study might be a contributing factor to this difference. It is also interesting to note that the share of old-growth forest species occurring in other forest uses is always higher for butterflies than dung beetles, possibly due to their higher mobility. We expect that the majority of insect species that we found uniquely in plantations are species adapted to the conditions found in open habitats (Gardner et al., 2008; Hamer et al., 2003).

The abundance differences in the two insect taxa analysed showed contrasting trends. Dung beetle abundance decreased gradually from old-growth forests to moderate use areas (BN: −9.9%; RIL: −17.9%) and dropped markedly in plantations (−58.0%). This is in line with results for the same region (Gardner et al., 2008), but differs from other studies (Berry et al., 2010; Nichols et al., 2007). Gardner et al. (2008) point to differences in biogeographical context and landscape-level effects between different studies as possible explanations for the discrepancy. Tree abundance slightly decreased (−12.8%) in RIL areas, an expected consequence of timber harvesting operations. Butterflies were considerably more abundant in BN areas (+24.3%) than in old-growth forest, but slightly less abundant in RIL areas (−5.1%). Their abundance more than doubled in plantations in relation to old-growth forests (+141.3%), while in a previous study for the same region they more than quadrupled (Barlow et al., 2007b). This was mostly driven by a few species of subfamilies Nymphalinae and Satyrinae that became hyper-abundant in plantations, due to their high tolerance to disturbance (Barlow et al., 2007b; Fermon et al., 2005).

### Congruence among taxa

Several multitaxon studies have considered potential cross-taxa congruence to assess to which degree one taxon can accurately predict the responses of multiple others (e.g., Barlow et al., 2007a; Edwards et al., 2014; Kremer, 1992). However, idiosyncratic responses to disturbance tend to deter generalisations (Gardner et al., 2009). Even higher taxonomic levels, such as butterfly subfamilies, may exhibit different responses (Barlow et al., 2007b; Hamer et al., 2003). Nevertheless, our results here point towards trees being the best indicator of all the taxa analysed, as it is the only taxon whose variations in richness, abundance and diversity are significantly correlated with all the other taxa analysed (Table 2). This is expected since both dung beetles and butterflies respond to changes in vegetation structure (Hamer et al., 2003; Gardner et al., 2008). Trees have been identified as good indicators of ecological change in other studies (Bobo et al., 2006; Philpott et al., 2008; Schulze et al., 2004), although they are not necessarily better indicators than other taxa (Barlow et al., 2007a; Kessler et al., 2009). The positive correlation between dung beetle and tree richness was identified in a previous study in the same region (Barlow et al., 2007a). Additionally, here we also report significant associations for abundance between trees and the insect taxa (with contrasting directions) and positive associations between tree and butterfly richness, as well as between the diversity of trees and both insect taxa (Table 2).

Seasonality effects might lead to low annual intra-taxon congruence in biodiversity sampling (Hamer et al., 2005a; Hamer et al., 2005b). Here, we find no evidence of seasonality in dung beetles across all forest uses, but both butterfly richness and abundance were significantly higher in the wet season. This variation might reflect natural inter-annual variation in community dynamics (Beiroz et al., 2017) but can also be related to differences in the community structure of each forest use (Barlow et al., 2007a). Indeed, the increased butterfly abundance in the wetter season registered here was mostly driven by a few species of Satyrinae (e.g., *Paryphthimoides* sp.) and Nymphalinae (e.g., *Hamadryas feronia*) in plantations that were rare or absent in the other forest uses.

### Caveats and limitations

It is relevant to highlight that the results presented here apply to a human-modified landscape containing large pools of old-growth forest. Therefore, extrapolating these results to landscapes under larger scale intensification processes should be avoided, due to potentially differentiated impacts of fragmentation and spill-over effects (Gardner et al., 2008; Korasaki et al., 2013; Nichols et al., 2007). Possible time lags in responses to forest use change also prevent conclusions regarding the long-term stability of the communities sampled ( Hautier et al., 2015).

While we consider the data collected representative of forest use conditions in the study area, the results should be interpreted with caution. Further sampling could particularly clarify the similarities and differences between communities in reduced impact logging and Brazil nut extraction areas. Still, the levels of species richness found here compare reasonably well to those found in previous studies in the same region with greater sampling efforts (Barlow et al., 2007a; Gardner et al., 2008; Sullivan et al., 2017). We suggest the reported differences in species richness and abundance are a consequence of variation in forest use intensity. Nevertheless, we cannot exclude the possibility that the patterns reported are being driven by certain unknown natural biotic or abiotic factors. The forest uses analysed are likely to be associated with varying pressures caused by factors such as logging, hunting, and fire occurrence, which indeed greatly affect natural communities in tropical forests (Barlow et al., 2016; Brodie et al., 2015). However, this study was not designed to isolate the individual effects of different disturbance factors within the forest use categories analysed.

### Implications for forest management

This study analyses forest uses with widespread occurrence in the Amazon region. It demonstrates that both reduced-impact logging and Brazil nut areas can have high conservation value. In fact, both these moderate forest uses seem to retain communities closer to those found in old-growth forests than the secondary forests that also occur within the same landscape (Barlow et al., 2007a; Gardner et al., 2008). This implies that the allocation of more resources to the promotion and improvement of moderate forest use regimes that prevent deforestation on the long term can benefit conservation at landscape scale. Biodiversity-friendly forest uses can increase the likelihood of attaining sustainable landscapes where people and forests are able to coexist on the long-term (Melo et al., 2013) and can also facilitate the effective implementation of Brazilian law (Law 12.651/2012), which requires that up to 80% of every rural property in the Amazon preserves its forest cover. Integral protection areas are fundamental for forest conservation (Gray et al., 2016), including within human-modified landscapes, where clusters of undisturbed forest can increase system resilience and preserve healthy species pools that can recolonise regenerating forests (Melo et al., 2013; Oliver et al., 2015). Nevertheless, sustainable use forests can still be relevant elements of extended networks of protected areas, providing viable corridors for movement between undisturbed areas or functioning as buffers that prevent forest encroachment (Bhagwat et al., 2008; McNeely & Schroth, 2006).

In north-eastern Amazonia, biodiversity-rich landscapes prevail for now. But there are indications that industrialised land uses will soon increase pressure on its forests, which in the absence of effective regulations and incentives that accommodate the needs of different stakeholders can, on the long term, lead to a repetition of deforestation patterns observed elsewhere (Melo et al., 2013; Soares-Filho et al., 2006). With more than two thirds of its territory protected, Amapá state is in a good position to trial a development model better suited to reconcile economic and conservation priorities through the prioritisation of biodiversity-based product value chains (Nobre et al., 2016). This will require landscape-level decision making, as well as further research, that acknowledges the multifunctionality of local socio-ecological systems (Cordingley et al., 2015; Reyers et al., 2013).

## Conclusion

This study demonstrates that increased forest use intensity is likely to cause negative effects on the communities of trees, dung beetles, and fruit-feeding butterflies of eastern Amazon. It therefore highlights the importance of preserving old-growth forests. Nevertheless, it also shows that biodiversity loss under extractive forest uses that introduce moderate disturbance can be relatively low, when integrated in a landscape matrix with a substantial share of old-growth forest. The two moderate uses analysed here, Brazil nut extraction and reduced impact logging, both hold substantial conservation value for the taxa studied and are able to retain communities that are relatively similar to those in old-growth forests, while intensive eucalyptus plantations have a higher impact on biodiversity. Results also confirm that studies considering multiple taxa and biodiversity metrics are more likely to provide a comprehensive perspective of how communities respond to disturbance in tropical forests. This study provides evidence to support safeguarding a healthy matrix of old-growth forest in forest management decisions and taking advantage of the potential of moderate forest uses to reconcile economic and nature conservation priorities.

##  Supplemental Information

10.7717/peerj.8486/supp-1 Supplemental Information 1 Supplemental Information Figure S1: Individual-based species accumulation curves.
Table S1: List of the species sampled per taxon and corresponding abundance data disaggregated per forest use.
Table S2: Results for the models y forest + sand + slope + elevation, where y corresponds to species richness or abundance of each sampled taxa.

## Additional Information and Declarations

### Field Study Permissions

The following information was supplied relating to field study approvals (i.e., approving body and any reference numbers): Fieldwork was authorised by Instituto Chico Mendes de Conservação da Biodiversidade (process number: 56120-1) and Conselho Nacional de Desenvolvimento Científico e Tecnológico (process number: 01300.001010/2016-16).

### Data Availability

The following information was supplied regarding data availability: The data is available at the UCL Discovery repository: Milheiras, S; (2019) Patterns of biodiversity response along a gradient of forest use in Eastern Amazonia, Brazil. [Dataset]. UCL Division of Biosciences: London, UK. http://discovery.ucl.ac.uk/10070652.
