# Supplementary material for: Patterns of biodiversity response along a gradient of forest use in Eastern Amazonia, Brazil
Source: PeerJ. 2020 Feb 11;8:e8486. doi: 10.7717/peerj.8486 (PMC7020811; doi:10.7717/peerj.8486)
Supplement: Supplemental Information 1 — Figure S1: Individual-based species accumulation curves. Table S1: List of the species sampled per taxon and corresponding abundance data disaggregated per forest use. Table S2: Results for the models y forest + sand + slope + elevation, where y corresponds to species richness or abundance of each sampled taxa. [file peerj-08-8486-s001.docx]

**Patterns of biodiversity response along a gradient of forest use in Eastern Amazonia, Brazil**

Sérgio G. Milheiras^1,5^, Marcelino Guedes^2^, Fernando A.B. Barbosa^3^, Perseu Aparício^4^, Georgina M. Mace^1^

^1^ Centre for Biodiversity and Environment Research, University College London, London, United Kingdom

^2^ EMBRAPA-Amapá, Macapá-AP, Brazil

^3^ Instituto de Ciências Biológicas, Universidade Federal do Pará, Belém-PA, Brazil

^4^ Universidade Estadual do Amapá, Macapá-AP, Brazil

^5^ School of Natural and Environmental Sciences, Newcastle University, Newcastle upon Tyne, United Kingdom

**Supplemental Information**

[**Figure S1:** Individual-based species accumulation curves. 2](#_Toc19868591)

[**Table S1:** List of the species sampled per taxon and forest use. 3](#_Toc19868592)

[**Table S2:** Results for the models y ~ forest + sand + slope + elevation, where y corresponds to either species richness, abundance or diversity of the three taxa sampled. 11](#_Toc19868593)


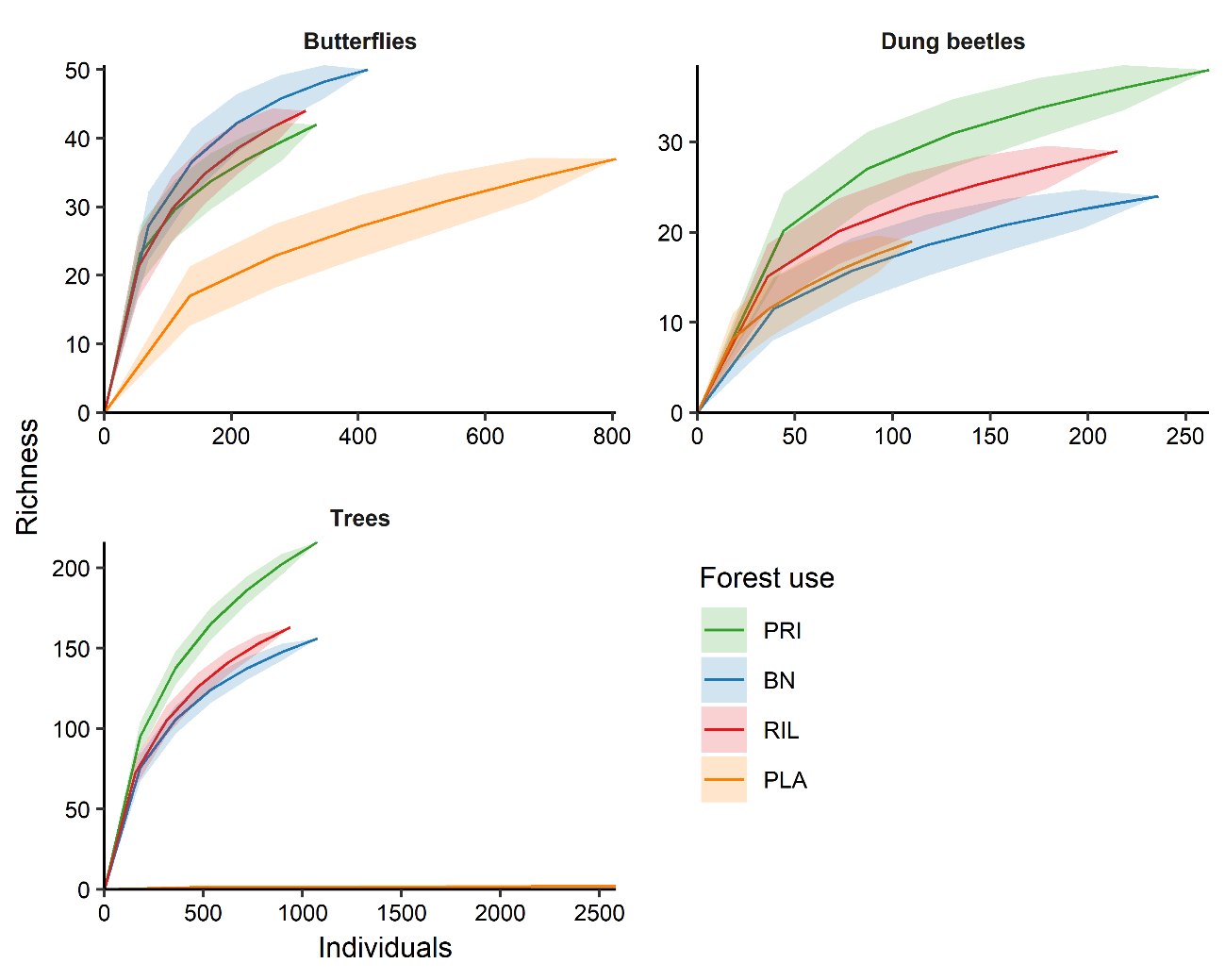


## Figure S1: Individual-based species accumulation curves, per forest use, for sampled butterflies, dung beetles, and trees. PRI: old-growth forest; BN: Brazil nut extraction areas; RIL: reduced impact logging areas; and PLA: eucalyptus plantations. Shaded areas correspond to the 95% confidence intervals for each curve.

## Table S1: List of the species sampled per taxon and corresponding abundance data disaggregated per forest use. The p-values shown were calculated with a Kruskal-Wallis test to determine if abundance levels between forest uses are significantly different for each species.

A) Dung beetles

| Dung beetles | BN | RIL | PLA | PRI | pvalue |
| --- | --- | --- | --- | --- | --- |
| *Ateuchus* aff. *connexus* | 11 | 29 | 1 | 6 | **0.007** |
| *Ateuchus* aff. *murrayi* | 3 | 21 | 0 | 0 | 0.079 |
| *Ateuchus irinus* | 34 | 0 | 0 | 1 | 0.065 |
| *Ateuchus pauki* | 0 | 1 | 0 | 0 | 0.392 |
| *Ateuchus* sp.1 | 0 | 0 | 0 | 1 | 0.392 |
| *Ateuchus* sp.2 | 2 | 0 | 6 | 0 | 0.262 |
| *Ateuchus* sp.3 | 1 | 0 | 0 | 0 | 0.392 |
| *Ateuchus* sp.4 | 1 | 0 | 0 | 0 | 0.392 |
| *Ateuchus* sp.5 | 3 | 4 | 0 | 0 | **0.033** |
| *Ateuchus* sp.6 | 14 | 0 | 0 | 0 | **0.02** |
| *Canthidium* aff. *deyrollei* | 1 | 0 | 0 | 13 | 0.059 |
| *Canthidium* aff. *lentum* | 0 | 0 | 14 | 0 | **0.02** |
| *Canthidium* sp.1 | 2 | 0 | 0 | 1 | 0.26 |
| *Canthidium* sp.2 | 0 | 0 | 0 | 6 | 0.099 |
| *Canthidium* sp.3 | 0 | 2 | 0 | 0 | 0.392 |
| *Canthidium* sp.4 | 21 | 1 | 0 | 8 | **0.02** |
| *Canthidium* sp.5 | 1 | 1 | 0 | 0 | 0.554 |
| *Canthidium* sp.6 | 1 | 0 | 0 | 0 | 0.392 |
| *Canthon bicolor* | 5 | 0 | 0 | 4 | 0.057 |
| *Canthon scrutator* | 0 | 0 | 4 | 0 | 0.392 |
| *Canthon simulans* | 0 | 0 | 13 | 0 | 0.1 |
| *Canthon subhyalinus* | 0 | 0 | 2 | 0 | 0.392 |
| *Canthon triangularis* | 0 | 7 | 1 | 10 | 0.108 |
| *Coprophanaeus jasius* | 0 | 0 | 1 | 0 | 0.392 |
| *Coprophanaeus lancifer* | 0 | 8 | 0 | 2 | **0.02** |
| *Deltochilum (Deltohyboma)* sp.1 | 0 | 1 | 29 | 1 | **0.032** |
| *Deltochilum (Deltohyboma)* sp.2 | 0 | 12 | 0 | 0 | **0** |
| *Deltochilum carinatum* | 0 | 0 | 0 | 2 | 0.392 |
| *Deltochilum icarus* | 1 | 5 | 0 | 6 | 0.09 |
| *Deltochilum orbiculare* | 0 | 1 | 0 | 6 | 0.235 |
| *Deltochilum septemstriatum* | 7 | 4 | 0 | 3 | 0.118 |
| *Dichotomius* aff. *lucasi* | 52 | 8 | 2 | 19 | 0.056 |
| *Dichotomius apicalis* | 0 | 0 | 0 | 3 | 0.1 |
| *Dichotomius boreus* | 2 | 43 | 1 | 24 | **0.003** |
| *Dichotomius imitator* | 0 | 0 | 1 | 0 | 0.392 |
| *Dichotomius latilobatus* | 0 | 0 | 1 | 0 | 0.392 |
| *Dichotomius mamillatus* | 0 | 0 | 0 | 9 | 0.392 |
| *Dichotomius roberti* | 0 | 0 | 0 | 2 | 0.099 |
| *Dichotomius subaeneus* | 0 | 1 | 0 | 0 | 0.392 |
| *Dichotomius worontzowi* | 4 | 2 | 6 | 5 | 0.933 |
| *Eurysternus atrosericus* | 0 | 8 | 1 | 5 | **0.021** |
| *Eurysternus balachowskyi* | 0 | 1 | 0 | 1 | 0.554 |
| *Eurysternus caribaeus* | 1 | 16 | 2 | 12 | 0.057 |
| *Eurysternus foedus* | 0 | 1 | 0 | 1 | 0.554 |
| *Eurysternus hamaticollis* | 0 | 6 | 0 | 4 | 0.098 |
| *Eurysternus hypocrita* | 0 | 0 | 0 | 8 | **0.02** |
| *Eurysternus vastiorum* | 0 | 0 | 0 | 1 | 0.392 |
| *Ontherus carinifrons* | 0 | 0 | 22 | 1 | 0.248 |
| *Onthophagus* aff. *bidentatus* | 1 | 2 | 0 | 7 | 0.411 |
| *Onthophagus* aff. *clypeatus* | 0 | 0 | 0 | 1 | 0.392 |
| *Onthophagus* aff. *haemathopus* | 4 | 15 | 0 | 11 | 0.055 |
| *Onthophagus* aff. *hircus* | 0 | 0 | 0 | 1 | 0.392 |
| *Onthophagus onthochromus* | 0 | 0 | 1 | 0 | 0.392 |
| *Oxysternon durantoni* | 0 | 0 | 0 | 2 | 0.099 |
| *Oxysternon festivum* | 2 | 8 | 2 | 33 | 0.168 |
| *Phanaeus chalcomelas* | 0 | 0 | 0 | 7 | 0.1 |
| *Sulcophanaeus faunus* | 0 | 1 | 0 | 1 | 0.554 |
| *Trichillum pauliani* | 0 | 1 | 0 | 0 | 0.392 |
| *Uroxys* sp. | 62 | 5 | 0 | 34 | **0.002** |

B) Butterflies

| Butterflies | BN | RIL | PLA | PRI | pvalue |
| --- | --- | --- | --- | --- | --- |
| *Amphidecta calliomma* | 2 | 0 | 0 | 0 | 0.099 |
| *Archaeoprepona amphimachus* | 4 | 1 | 1 | 0 | 0.477 |
| *Archaeoprepona demophon* | 24 | 14 | 2 | 16 | **0.015** |
| *Archaeoprepona licomedes* | 3 | 0 | 0 | 4 | 0.207 |
| *Archaeoprepona meander* | 1 | 2 | 0 | 0 | 0.26 |
| *Bia actorion* | 13 | 6 | 0 | 24 | **0.018** |
| *Caeruleuptychia* aff. *coelestis* | 0 | 1 | 0 | 0 | 0.392 |
| *Caeruleuptychia brixius* | 1 | 0 | 0 | 0 | 0.392 |
| *Caeruleuptychia urania* | 0 | 1 | 0 | 0 | 0.392 |
| *Caligo brasiliensis* | 4 | 2 | 0 | 1 | 0.211 |
| *Caligo euphorbus* | 14 | 2 | 0 | 3 | **0.009** |
| *Catoblepia berecynthia* | 20 | 4 | 1 | 12 | **0.004** |
| *Catoblepia versitincta* | 1 | 0 | 0 | 0 | 0.392 |
| *Catoblepia xanthus* | 10 | 5 | 0 | 1 | **0.028** |
| *Catonephele acontius* | 7 | 65 | 0 | 13 | **0.003** |
| *Catonephele numilia* | 0 | 2 | 1 | 0 | 0.26 |
| *Chloreuptychia agatha* | 4 | 0 | 0 | 1 | 0.07 |
| *Chloreuptychia chlorimene* | 0 | 3 | 0 | 0 | 0.1 |
| *Cissia myncea* | 0 | 0 | 1 | 1 | 0.554 |
| *Cissia penelope* | 0 | 0 | 10 | 0 | **0** |
| *Cissia terrestris* | 1 | 7 | 6 | 3 | 0.574 |
| *Colobura* cf. *annulata* | 4 | 2 | 1 | 5 | 0.654 |
| *Colobura dirce* | 11 | 29 | 41 | 15 | 0.115 |
| *Erichthodes antonina* | 0 | 0 | 0 | 3 | 0.1 |
| *Eryphanis automedon* | 4 | 1 | 0 | 10 | **0.001** |
| *Fountainea ryphea* | 0 | 0 | 2 | 0 | 0.099 |
| *Hamadryas amphinome* | 0 | 0 | 6 | 0 | **0.02** |
| *Hamadryas arinome* | 0 | 1 | 1 | 0 | 0.554 |
| *Hamadryas chloe* | 0 | 0 | 0 | 1 | 0.392 |
| *Hamadryas februa* | 0 | 1 | 241 | 1 | **0** |
| *Hamadryas feronia* | 0 | 1 | 96 | 0 | **0.001** |
| *Hamadryas iphthime* | 0 | 0 | 3 | 0 | **0.02** |
| *Hamadryas laodamia* | 0 | 0 | 1 | 0 | 0.392 |
| *Hermeuptychia* sp. | 0 | 0 | 30 | 1 | **0.002** |
| *Historis odius* | 0 | 0 | 3 | 0 | 0.1 |
| *Hypna clytemnestra* | 4 | 5 | 0 | 4 | 0.223 |
| *Junonia evarete* | 0 | 0 | 1 | 0 | 0.392 |
| *Magneuptychia libye* | 0 | 0 | 22 | 0 | **0** |
| *Magneuptychia newtonii* | 0 | 0 | 8 | 0 | **0.003** |
| *Magneuptychia tricolor* | 4 | 0 | 0 | 1 | 0.07 |
| *Memphis* cf. *acidalia* | 2 | 5 | 8 | 1 | 0.237 |
| *Memphis laertes* | 2 | 0 | 0 | 0 | 0.392 |
| *Memphis oenomais* | 1 | 0 | 1 | 0 | 0.554 |
| *Memphis polycarmes* | 2 | 0 | 0 | 1 | 0.26 |
| *Mesoprepona pheridamas* | 33 | 6 | 0 | 15 | **0.014** |
| *Morpho achilles* | 4 | 4 | 1 | 5 | 0.497 |
| *Morpho deidamia* | 1 | 0 | 0 | 0 | 0.392 |
| *Morpho helenor* | 5 | 8 | 1 | 6 | 0.067 |
| *Morpho menelaus* | 1 | 0 | 0 | 0 | 0.392 |
| *Nessaea obrinus* | 45 | 1 | 1 | 32 | **0.001** |
| *Opsiphanes cassiae* | 2 | 1 | 1 | 0 | 0.513 |
| *Opsiphanes cassina* | 2 | 0 | 0 | 0 | 0.392 |
| *Opsiphanes invirae* | 3 | 3 | 0 | 1 | 0.156 |
| *Opsiphanes quiteria* | 1 | 0 | 0 | 4 | 0.234 |
| *Pareuptychia lydia* | 9 | 9 | 0 | 18 | 0.071 |
| *Pareuptychia ocirrhoe* | 10 | 15 | 2 | 15 | 0.113 |
| *Paryphthimoides* sp. | 0 | 0 | 131 | 1 | **0** |
| *Posttaygetis penelea* | 4 | 0 | 0 | 0 | **0.02** |
| *Prepona claudina* | 0 | 1 | 0 | 0 | 0.392 |
| *Prepona narcissus* | 0 | 0 | 0 | 1 | 0.392 |
| *Prepona rhenea* | 3 | 2 | 0 | 0 | 0.088 |
| *Pseudodebis celia* | 1 | 0 | 0 | 0 | 0.392 |
| *Pseudodebis valentina* | 15 | 0 | 0 | 1 | **0** |
| *Taygetina kerea* | 0 | 1 | 0 | 0 | 0.392 |
| *Taygetis cleopatra* | 37 | 46 | 1 | 21 | **0.003** |
| *Taygetis echo* | 29 | 8 | 0 | 15 | **0.002** |
| *Taygetis laches* | 12 | 2 | 162 | 26 | **0.029** |
| *Taygetis larua* | 2 | 0 | 0 | 0 | 0.099 |
| *Taygetis leuctra* | 0 | 1 | 0 | 0 | 0.392 |
| *Taygetis mermeria* | 4 | 0 | 0 | 6 | **0.041** |
| *Taygetis rufomarginata* | 4 | 6 | 5 | 4 | 0.88 |
| *Taygetis sosis* | 0 | 4 | 1 | 0 | 0.248 |
| *Taygetis zippora* | 34 | 22 | 0 | 23 | **0.003** |
| *Temenis laothoe* | 0 | 1 | 2 | 0 | 0.26 |
| *Tigridia acesta* | 7 | 5 | 0 | 13 | **0.01** |
| *Yphthimoides renata* | 0 | 2 | 9 | 1 | 0.062 |
| *Zaretis isidora* | 2 | 0 | 1 | 0 | 0.26 |
| *Zaretis itys* | 2 | 9 | 1 | 4 | 0.054 |

C) Trees

| Trees | BN | RIL | PLA | PRI | pvalue |
| --- | --- | --- | --- | --- | --- |
| *Acosmium nitens* | 0 | 3 | 0 | 1 | 0.248 |
| *Alexa grandiflora* | 0 | 0 | 0 | 1 | 0.392 |
| *Amanoa guianensis* | 1 | 2 | 0 | 3 | 0.433 |
| *Anacardium giganteum* | 0 | 1 | 0 | 1 | 0.554 |
| *Anaxagorea dolichocarpa* | 1 | 0 | 0 | 0 | 0.392 |
| *Aniba* sp. | 4 | 4 | 0 | 8 | **0.048** |
| *Antonia ovata* | 2 | 3 | 0 | 0 | 0.088 |
| *Apeiba burchellii* | 13 | 7 | 0 | 3 | **0.004** |
| *Aspidosperma carapanauba* | 2 | 3 | 0 | 2 | 0.295 |
| *Aspidosperma desmanthum* | 0 | 0 | 0 | 1 | 0.392 |
| *Aspidosperma eteanum* | 10 | 1 | 0 | 6 | 0.09 |
| *Aspidosperma megalocarpon* | 0 | 0 | 0 | 1 | 0.392 |
| *Aspidosperma* sp. | 9 | 17 | 0 | 15 | **0.013** |
| *Astronium gracile* | 0 | 5 | 0 | 5 | **0.032** |
| *Bagassa guianensis* | 0 | 0 | 0 | 1 | 0.392 |
| *Batesia floribunda* | 1 | 0 | 0 | 0 | 0.392 |
| *Bauhinia* sp. | 1 | 6 | 0 | 0 | 0.553 |
| *Beilschmiedia* sp. | 3 | 0 | 0 | 6 | 0.054 |
| *Bellucia dichotoma* | 4 | 1 | 0 | 14 | 0.409 |
| *Bertholletia excelsa* | 10 | 0 | 0 | 4 | **0.024** |
| *Bombacopsis nervosa* | 0 | 0 | 0 | 1 | 0.392 |
| *Bowdichia nitida* | 6 | 3 | 0 | 7 | 0.208 |
| *Brosimum parinarioides* | 5 | 1 | 0 | 4 | 0.216 |
| *Buchenavia grandis* | 0 | 2 | 0 | 0 | 0.392 |
| *Buchenavia parvifolia* | 1 | 0 | 0 | 3 | 0.553 |
| *Buchenavia* sp. | 0 | 2 | 0 | 2 | 0.286 |
| *Byrsonima aerugo* | 0 | 0 | 0 | 3 | 0.1 |
| *Capirona decorticans* | 0 | 1 | 0 | 0 | 0.392 |
| *Carapa guianensis* | 4 | 0 | 0 | 3 | 0.099 |
| *Caryocar glabrum* | 0 | 1 | 0 | 0 | 0.392 |
| *Caryocar villosum* | 5 | 3 | 0 | 4 | 0.172 |
| *Casearia javitensis* | 0 | 1 | 0 | 0 | 0.392 |
| *Cecropia obtusa* | 5 | 1 | 1 | 4 | 0.197 |
| *Cecropia* sp. | 2 | 0 | 0 | 4 | 0.201 |
| *Cedrela odorata* | 0 | 0 | 0 | 2 | 0.099 |
| *Cedrelinga* sp. | 1 | 0 | 0 | 0 | 0.392 |
| *Ceiba pentandra* | 1 | 0 | 0 | 2 | 0.553 |
| *Chamaecrista bahiae* | 3 | 0 | 0 | 3 | 0.099 |
| *Chaunochiton kappleri* | 0 | 0 | 0 | 1 | 0.392 |
| *Chimarrhis turbinata* | 6 | 10 | 0 | 4 | 0.066 |
| *Conceveiba guianensis* | 2 | 1 | 0 | 1 | 0.776 |
| *Conceveiba martiana* | 6 | 0 | 0 | 4 | 0.092 |
| *Copaifera martii* | 0 | 2 | 0 | 0 | 0.099 |
| *Couepia robusta* | 0 | 1 | 0 | 0 | 0.392 |
| *Couma guianensis* | 0 | 1 | 0 | 1 | 0.554 |
| *Couratari pulchra* | 3 | 3 | 0 | 2 | 0.467 |
| *Couroupita* sp. | 0 | 1 | 0 | 0 | 0.392 |
| *Dacryodes nitens* | 1 | 0 | 0 | 3 | 0.248 |
| *Dialium guianense* | 3 | 3 | 0 | 0 | 0.053 |
| *Didymopanax morototoni* | 11 | 0 | 0 | 7 | **0.004** |
| *Dimorphandra multiflora* | 0 | 0 | 0 | 1 | 0.392 |
| *Dinizia excelsa* | 0 | 6 | 0 | 0 | **0** |
| *Diospyros santaremnensis* | 0 | 3 | 0 | 2 | 0.205 |
| *Diospyros* sp. | 0 | 0 | 0 | 4 | 0.099 |
| *Diplotropis purpurea* | 1 | 1 | 0 | 6 | 0.134 |
| *Diplotropis racemosa* | 1 | 2 | 0 | 1 | 0.513 |
| *Dipteryx magnifica* | 0 | 0 | 0 | 1 | 0.392 |
| *Dipteryx odorata* | 10 | 5 | 0 | 13 | **0.038** |
| *Drypetes variabilis* | 0 | 2 | 0 | 1 | 0.553 |
| *Duguetia surinamensis* | 0 | 1 | 0 | 2 | 0.26 |
| *Duroia macrophylla* | 1 | 0 | 0 | 4 | 0.248 |
| *Duroia* sp. | 2 | 0 | 0 | 3 | 0.274 |
| *Dussia discolor* | 0 | 2 | 0 | 0 | 0.392 |
| *Ecclinusa abbreviata* | 1 | 0 | 0 | 0 | 0.392 |
| *Endopleura uchi* | 1 | 4 | 0 | 1 | 0.477 |
| *Enterolobium schomburgkii* | 1 | 3 | 0 | 0 | 0.248 |
| *Enterolobium* sp. | 1 | 0 | 0 | 2 | 0.553 |
| *Eriotheca globosa* | 2 | 0 | 0 | 0 | 0.099 |
| *Erisma laurifolium* | 0 | 1 | 0 | 0 | 0.392 |
| *Erisma* sp. | 0 | 1 | 0 | 0 | 0.392 |
| *Eschweilera amazonica* | 0 | 3 | 0 | 4 | 0.286 |
| *Eschweilera coriacea* | 0 | 0 | 0 | 1 | 0.392 |
| *Eschweilera obversa* | 0 | 1 | 0 | 0 | 0.392 |
| *Eschweilera odora* | 27 | 8 | 0 | 13 | **0.011** |
| *Eschweilera* sp. | 0 | 13 | 0 | 1 | **0.002** |
| *Eucalyptus* sp. | 0 | 0 | 2583 | 0 | **0** |
| *Eugenia patrisii* | 0 | 0 | 0 | 1 | 0.392 |
| *Ferdinandusa paraensis* | 0 | 2 | 0 | 1 | 0.26 |
| *Ficus nymphaeifolia* | 0 | 2 | 0 | 1 | 0.26 |
| *Geissospermum sericeum* | 0 | 45 | 0 | 16 | **0.001** |
| *Glycydendron* sp. | 0 | 1 | 0 | 0 | 0.392 |
| *Goupia glabra* | 8 | 8 | 0 | 8 | 0.184 |
| *Guarania* sp. | 0 | 0 | 0 | 1 | 0.392 |
| *Guarea silvatica* | 0 | 0 | 0 | 12 | **0.02** |
| *Guarea* sp. | 0 | 0 | 0 | 15 | **0.02** |
| *Guatteria poeppigiana* | 9 | 3 | 0 | 9 | 0.063 |
| *Guatteria* sp. | 0 | 11 | 0 | 1 | **0.01** |
| *Guazuma ulmifolia* | 0 | 1 | 0 | 1 | 0.554 |
| *Gustavia augusta* | 65 | 0 | 0 | 4 | **0.005** |
| *Hebepetalum humiriifolium* | 12 | 0 | 0 | 10 | **0.009** |
| *Helicostylis* sp. | 0 | 9 | 0 | 5 | 0.098 |
| *Herrania mariae* | 0 | 2 | 0 | 1 | 0.553 |
| *Hevea brasiliensis* | 2 | 0 | 0 | 0 | 0.392 |
| *Himatanthus sucuuba* | 1 | 0 | 0 | 1 | 0.554 |
| *Hirtella bicornis* | 1 | 0 | 0 | 2 | 0.553 |
| *Hirtella piresii* | 12 | 1 | 0 | 3 | 0.051 |
| *Hirtella* sp. | 7 | 8 | 0 | 1 | **0.031** |
| *Hymenaea courbaril* | 1 | 1 | 0 | 0 | 0.554 |
| *Hymenaea intermedia* | 0 | 1 | 0 | 0 | 0.392 |
| *Hymenaea parvifolia* | 0 | 1 | 0 | 1 | 0.554 |
| *Hymenolobium excelsum* | 0 | 1 | 0 | 0 | 0.392 |
| *Hymenolobium sericeum* | 0 | 0 | 0 | 1 | 0.392 |
| *Inga acrocephala* | 0 | 0 | 0 | 2 | 0.099 |
| *Inga alba* | 4 | 0 | 0 | 15 | **0.02** |
| *Inga cayennensis* | 10 | 0 | 0 | 5 | **0.003** |
| *Inga gracilifolia* | 9 | 0 | 0 | 1 | **0.012** |
| *Inga heterophylla* | 6 | 0 | 0 | 15 | **0.01** |
| *Inga negrensis* | 5 | 0 | 0 | 1 | 0.07 |
| *Inga paraensis* | 0 | 5 | 0 | 2 | 0.078 |
| *Inga rubiginosa* | 1 | 2 | 0 | 0 | 0.26 |
| *Inga splendens* | 3 | 0 | 0 | 21 | 0.054 |
| *Inga subsericantha* | 0 | 0 | 0 | 7 | **0.02** |
| *Inga tarapotensis* | 1 | 0 | 0 | 0 | 0.392 |
| *Iryanthera juruensis* | 24 | 0 | 0 | 21 | **0.001** |
| *Iryanthera* sp. | 4 | 0 | 0 | 2 | 0.091 |
| *Jacaranda copaia* | 0 | 2 | 0 | 52 | 0.205 |
| *Jacaratia* sp. | 0 | 0 | 0 | 1 | 0.392 |
| *Lacmellea gracilis* | 0 | 0 | 0 | 1 | 0.392 |
| *Lacunaria spruceana* | 0 | 0 | 0 | 1 | 0.392 |
| *Laetia procera* | 13 | 10 | 0 | 6 | 0.158 |
| *Lecythis corrugata* | 0 | 1 | 0 | 0 | 0.392 |
| *Lecythis lurida* | 0 | 0 | 0 | 2 | 0.099 |
| *Lecythis poiteaui* | 5 | 0 | 0 | 2 | **0.022** |
| *Lecythis* sp. | 15 | 0 | 0 | 4 | **0.004** |
| *Lecythis usitata* | 10 | 3 | 0 | 1 | **0.013** |
| *Licania heteromorpha* | 3 | 0 | 0 | 3 | 0.053 |
| *Licania laevigata* | 0 | 0 | 0 | 2 | 0.392 |
| *Licania latifolia* | 17 | 11 | 0 | 9 | **0.027** |
| *Licania micrantha* | 0 | 16 | 0 | 2 | **0.018** |
| *Licania robusta* | 0 | 3 | 0 | 0 | 0.1 |
| *Licania* sp. | 1 | 4 | 0 | 1 | 0.18 |
| *Licaria cannella* | 2 | 0 | 0 | 0 | 0.392 |
| *Luehea speciosa* | 1 | 0 | 0 | 1 | 0.554 |
| *Macoubea guianensis* | 6 | 0 | 0 | 3 | **0.007** |
| *Manilkara bidentata* | 0 | 13 | 0 | 1 | **0.002** |
| *Manilkara huberi* | 1 | 16 | 0 | 6 | **0.002** |
| *Maquira guianensis* | 0 | 3 | 0 | 0 | 0.392 |
| *Maquira sclerophylla* | 44 | 17 | 0 | 19 | **0.005** |
| *Mezilaurus itauba* | 4 | 0 | 0 | 1 | 0.07 |
| *Mezilaurus lindaviana* | 9 | 1 | 0 | 3 | 0.064 |
| *Miconia guianensis* | 4 | 0 | 0 | 13 | **0.018** |
| *Miconia rosea* | 2 | 1 | 0 | 10 | **0.012** |
| *Miconia* sp. | 0 | 0 | 0 | 1 | 0.392 |
| *Miconia surinamensis* | 0 | 2 | 0 | 6 | 0.077 |
| *Micropholis mensalis* | 1 | 0 | 0 | 1 | 0.554 |
| *Minquartia guianensis* | 3 | 2 | 0 | 5 | 0.333 |
| *Mouriri brachyanthera* | 5 | 5 | 0 | 10 | **0.045** |
| *Mouriri brevipes* | 0 | 1 | 0 | 3 | 0.248 |
| *Mouriri collocarpa* | 9 | 5 | 0 | 5 | **0.012** |
| *Mouriri* sp. | 2 | 11 | 0 | 1 | **0.012** |
| *Myrcia fallax* | 0 | 1 | 0 | 1 | 0.554 |
| *Myrciaria floribunda* | 0 | 2 | 0 | 0 | 0.392 |
| *Neea constricta* | 0 | 1 | 0 | 1 | 0.554 |
| *Neea* sp. | 0 | 5 | 0 | 0 | 0.1 |
| *Nemaluma engleri* | 0 | 0 | 0 | 2 | 0.099 |
| *Ocotea amazonica* | 0 | 0 | 0 | 1 | 0.392 |
| *Ocotea douradensis* | 22 | 1 | 0 | 16 | **0.002** |
| *Ocotea* sp. | 0 | 1 | 0 | 1 | 0.554 |
| *Oenocarpus bacaba* | 6 | 16 | 0 | 15 | 0.085 |
| *Onychopetalum amazonicum* | 0 | 2 | 0 | 3 | 0.553 |
| *Ormosia coccinea* | 1 | 1 | 0 | 0 | 0.554 |
| *Ormosia coutinhoi* | 1 | 0 | 0 | 0 | 0.392 |
| *Ormosia paraensis* | 0 | 1 | 0 | 1 | 0.554 |
| *Osteophloeum platyspermum* | 2 | 0 | 0 | 1 | 0.26 |
| *Ouratea oliviformis* | 3 | 1 | 0 | 7 | 0.424 |
| *Paraprotium amazonicum* | 29 | 0 | 0 | 33 | **0.001** |
| *Parinari excelsa* | 5 | 2 | 0 | 11 | 0.056 |
| *Parkia oppositifolia* | 1 | 4 | 0 | 5 | 0.139 |
| *Parkia pendula* | 3 | 3 | 0 | 3 | 0.267 |
| *Parkia reticulata* | 0 | 0 | 0 | 1 | 0.392 |
| *Parkia ulei* | 0 | 0 | 0 | 2 | 0.099 |
| *Peltogyne paniculata* | 0 | 0 | 0 | 1 | 0.392 |
| *Peltogyne paradoxa* | 0 | 1 | 0 | 0 | 0.392 |
| *Pentaclethra macroloba* | 10 | 0 | 0 | 0 | 0.392 |
| *Persea jariensis* | 22 | 3 | 0 | 10 | 0.127 |
| *Piptadenia communis* | 1 | 6 | 0 | 4 | 0.202 |
| *Pithecellobium decandrum* | 1 | 6 | 0 | 4 | **0.033** |
| *Pithecellobium racemosum* | 0 | 18 | 0 | 4 | **0** |
| *Pithecellobium* sp. | 1 | 2 | 0 | 0 | 0.553 |
| *Platonia insignis* | 0 | 0 | 0 | 1 | 0.392 |
| *Platymiscium* sp. | 0 | 0 | 0 | 1 | 0.392 |
| *Pogonophora schomburgkiana* | 1 | 3 | 0 | 1 | 0.204 |
| *Pourouma* sp. | 3 | 1 | 0 | 8 | 0.125 |
| *Pourouma villosa* | 0 | 1 | 0 | 0 | 0.392 |
| *Pouteria amazonica* | 2 | 0 | 0 | 1 | 0.26 |
| *Pouteria bilocularis* | 9 | 10 | 0 | 7 | 0.071 |
| *Pouteria cladantha* | 0 | 8 | 0 | 0 | **0.02** |
| *Pouteria elegans* | 2 | 0 | 0 | 10 | 0.071 |
| *Pouteria jariensis* | 2 | 32 | 0 | 1 | **0** |
| *Pouteria krukovii* | 1 | 10 | 0 | 3 | 0.057 |
| *Pouteria laurifolia* | 0 | 3 | 0 | 3 | 0.099 |
| *Pouteria* sp. | 6 | 16 | 0 | 9 | 0.08 |
| *Pouteria spruceana* | 7 | 11 | 0 | 2 | **0.023** |
| *Pouteria torta* | 6 | 3 | 0 | 2 | 0.221 |
| *Prieurella* sp. | 0 | 1 | 0 | 1 | 0.554 |
| *Protium altsonii* | 6 | 0 | 0 | 3 | 0.204 |
| *Protium apiculatum* | 0 | 2 | 0 | 3 | 0.205 |
| *Protium decandrum* | 8 | 53 | 0 | 16 | **0.007** |
| *Protium giganteum* | 0 | 1 | 0 | 0 | 0.392 |
| *Protium heptaphyllum* | 11 | 0 | 0 | 20 | **0.009** |
| *Protium juruense* | 0 | 0 | 0 | 1 | 0.392 |
| *Protium krukoffii* | 0 | 1 | 0 | 0 | 0.392 |
| *Protium opacum* | 1 | 2 | 0 | 3 | 0.235 |
| *Protium pallidum* | 20 | 6 | 0 | 5 | 0.061 |
| *Protium paniculatum* | 2 | 3 | 0 | 1 | 0.513 |
| *Protium sagotianum* | 2 | 68 | 0 | 2 | 0.051 |
| *Protium* sp. | 83 | 17 | 0 | 57 | **0.005** |
| *Protium subserratum* | 0 | 16 | 0 | 0 | **0.02** |
| *Protium tenuifolium* | 0 | 5 | 0 | 2 | 0.205 |
| *Psychotria mapourioides* | 1 | 0 | 0 | 2 | 0.553 |
| *Pterocarpus rohrii* | 0 | 1 | 0 | 0 | 0.392 |
| *Qualea albiflora* | 2 | 1 | 0 | 8 | **0.045** |
| *Qualea* sp. | 0 | 6 | 0 | 3 | 0.054 |
| *Radlkoferella macrocarpa* | 2 | 5 | 0 | 2 | 0.239 |
| *Rheedia macrophylla* | 0 | 0 | 0 | 1 | 0.392 |
| *Rheedia* sp. | 11 | 0 | 0 | 0 | **0.003** |
| *Rinorea amapensis* | 2 | 0 | 0 | 0 | 0.392 |
| *Rinorea guianensis* | 0 | 1 | 0 | 8 | 0.07 |
| *Roupala montana* | 0 | 0 | 0 | 1 | 0.392 |
| *Sacoglottis amazonica* | 0 | 1 | 0 | 0 | 0.392 |
| *Sacoglottis guianensis* | 1 | 2 | 0 | 3 | 0.433 |
| *Sclerolobium melanocarpum* | 1 | 0 | 0 | 1 | 0.554 |
| *Sclerolobium melinonii* | 0 | 1 | 0 | 0 | 0.392 |
| *Simaba cedron* | 5 | 0 | 0 | 6 | 0.207 |
| *Simarouba amara* | 3 | 2 | 0 | 3 | 0.312 |
| *Sloanea grandis* | 0 | 2 | 0 | 1 | 0.26 |
| *Sloanea obtusa* | 0 | 3 | 0 | 0 | **0.02** |
| *Sloanea* sp. | 4 | 0 | 0 | 0 | **0.02** |
| *Socratea exorrhiza* | 3 | 0 | 0 | 0 | 0.392 |
| *Spondias mombin* | 0 | 0 | 0 | 2 | 0.392 |
| *Sterculia amazonica* | 1 | 0 | 0 | 0 | 0.392 |
| *Sterculia pilosa* | 11 | 1 | 0 | 11 | **0.003** |
| *Sterculia roseiflora* | 0 | 0 | 0 | 1 | 0.392 |
| *Stryphnodendron paniculatum* | 4 | 0 | 0 | 0 | 0.099 |
| *Stryphnodendron* sp. | 0 | 0 | 0 | 1 | 0.392 |
| *Styrax sieberi* | 0 | 0 | 0 | 1 | 0.392 |
| *Swartzia amazonica* | 0 | 0 | 0 | 1 | 0.392 |
| *Swartzia grandifolia* | 0 | 1 | 0 | 0 | 0.392 |
| *Swartzia panacoco* | 2 | 4 | 0 | 3 | 0.113 |
| *Swartzia polyphylla* | 0 | 0 | 0 | 1 | 0.392 |
| *Swartzia* sp. | 0 | 0 | 0 | 1 | 0.392 |
| *Symphonia globulifera* | 1 | 0 | 0 | 0 | 0.392 |
| *Syzygiopsis oppositifolia* | 0 | 0 | 0 | 1 | 0.392 |
| *Syzygiopsis* sp. | 0 | 4 | 0 | 4 | **0.023** |
| *Tabebuia impetiginosa* | 1 | 1 | 0 | 0 | 0.554 |
| *Tabebuia serratifolia* | 0 | 1 | 0 | 0 | 0.392 |
| *Tachigali alba* | 0 | 4 | 0 | 0 | 0.099 |
| *Tachigali myrmecophila* | 19 | 16 | 0 | 21 | **0.03** |
| *Tachigali* sp. | 2 | 8 | 0 | 11 | **0.049** |
| *Tapirira* sp. | 0 | 2 | 0 | 15 | 0.202 |
| *Tapura amazonica* | 1 | 0 | 0 | 0 | 0.392 |
| *Terminalia amazonia* | 0 | 1 | 0 | 0 | 0.392 |
| *Terminalia* sp. | 0 | 0 | 0 | 1 | 0.392 |
| *Tetragastris altissima* | 1 | 0 | 0 | 0 | 0.392 |
| *Tetragastris panamensis* | 56 | 16 | 0 | 16 | **0.016** |
| *Theobroma subincanum* | 72 | 3 | 0 | 17 | **0.023** |
| *Theobroma sylvestre* | 8 | 0 | 0 | 2 | **0.003** |
| *Thyrsodium guianense* | 0 | 7 | 0 | 1 | **0.002** |
| *Toulicia* sp. | 6 | 0 | 0 | 4 | 0.057 |
| *Tovomita cephalostigma* | 0 | 0 | 0 | 1 | 0.392 |
| *Trattinnickia burseraefolia* | 0 | 1 | 0 | 3 | 0.248 |
| *Trattinnickia rhoifolia* | 3 | 0 | 0 | 3 | 0.099 |
| *Trattinnickia* sp. | 0 | 1 | 0 | 1 | 0.554 |
| *Trichilia lecointei* | 2 | 0 | 0 | 3 | 0.205 |
| *Trichilia septentrionalis* | 0 | 4 | 0 | 5 | 0.098 |
| *Vantanea guianensis* | 1 | 0 | 0 | 0 | 0.392 |
| *Vantanea parviflora* | 0 | 6 | 0 | 0 | **0.003** |
| *Vatairea erythrocarpa* | 1 | 2 | 0 | 3 | 0.235 |
| *Vataireopsis speciosa* | 0 | 0 | 0 | 1 | 0.392 |
| *Virola calophylla* | 1 | 3 | 0 | 4 | 0.513 |
| *Virola flexuosa* | 4 | 7 | 0 | 1 | 0.072 |
| *Virola melinonii* | 4 | 5 | 0 | 0 | **0.005** |
| *Virola michelii* | 7 | 3 | 0 | 3 | 0.474 |
| *Virola* sp. | 0 | 0 | 0 | 1 | 0.392 |
| *Vismia cayennensis* | 3 | 0 | 0 | 0 | 0.392 |
| *Vismia* sp. | 0 | 0 | 0 | 2 | 0.099 |
| *Vochysia eximia* | 8 | 0 | 0 | 8 | **0.022** |
| *Vochysia guianensis* | 2 | 0 | 0 | 1 | 0.553 |
| *Vochysia maxima* | 0 | 1 | 0 | 0 | 0.392 |
| *Vochysia obscura* | 3 | 5 | 0 | 7 | 0.123 |
| *Vochysia paraensis* | 0 | 0 | 0 | 2 | 0.099 |
| *Vochysia splendens* | 5 | 0 | 0 | 0 | 0.392 |
| *Vochysia vismiifolia* | 1 | 0 | 0 | 0 | 0.392 |
| *Vouacapoua americana* | 0 | 79 | 0 | 9 | **0.001** |
| *Xylopia benthamii* | 14 | 0 | 0 | 13 | 0.089 |
| *Zanthoxylum regnellianum* | 1 | 0 | 0 | 0 | 0.392 |
| *Zygia latifolia* | 0 | 0 | 0 | 1 | 0.392 |

## Table S2: Results for the models y ~ forest + sand + slope + elevation, where y corresponds to either species richness, abundance or diversity of the three taxa sampled. ‘Forest’ is a categorical variable with four levels (PRI, BN, RIL, PLA), with PRI being the reference category. ‘Sand’ refers to the percentage of sand in the soil at each site. Significant p-values are shown in bold.

|  |  | Richness | | | | Abundance | | | | Diversity | | | |
| --- | --- | --- | --- | --- | --- | --- | --- | --- | --- | --- | --- | --- | --- |
| Taxa | **Variables** | **Coef.** | **S.Error** | **t-value** | **p-value** | **Coef.** | **S.Error** | **t-value** | **p-value** | **Coef.** | **S.Error** | **t-value** | **p-value** |
| Butter-flies | *Intercept* | 12.328 | 6.868 | 1.795 | 0.09 | 6.951 | 44.652 | 0.156 | 0.878 | 9.406 | 3.775 | 2.492 | **0.023** |
|  | *Forest: BN* | 4.689 | 2.201 | 2.131 | **0.048** | 3.631 | 14.309 | 0.254 | 0.803 | 2.051 | 1.21 | 1.695 | 0.108 |
|  | *Forest: RIL* | 1.913 | 2.72 | 0.703 | 0.491 | 0.937 | 17.683 | 0.053 | 0.958 | -1.713 | 1.495 | -1.146 | 0.268 |
|  | *Forest: PLA* | -4.457 | 2.617 | -1.703 | 0.107 | 69.718 | 17.014 | 4.098 | **0.001** | -8.526 | 1.439 | -5.927 | **0** |
|  | *Sand* | 0.01 | 0.005 | 2.004 | 0.061 | 0.05 | 0.033 | 1.525 | 0.146 | 0.024 | 0.028 | 0.85 | 0.407 |
|  | *Slope* | 0.165 | 0.206 | 0.801 | 0.434 | -0.927 | 1.339 | -0.693 | 0.498 | 0.137 | 0.113 | 1.21 | 0.243 |
|  | *Elevation* | 0.012 | 0.039 | 0.316 | 0.756 | 0.217 | 0.254 | 0.855 | 0.404 | 0.013 | 0.021 | 0.604 | 0.554 |
| Dung beetles | *Intercept* | 25.686 | 7.465 | 3.441 | **0.003** | 88.267 | 38.735 | 2.279 | **0.036** | 14.922 | 3.607 | 4.137 | **0.001** |
|  | *Forest: BN* | -3.137 | 2.392 | -1.311 | 0.207 | -5.573 | 12.413 | -0.449 | 0.659 | -1.969 | 1.156 | -1.703 | 0.107 |
|  | *Forest: RIL* | -2.232 | 2.956 | -0.755 | 0.461 | -25.269 | 15.34 | -1.647 | 0.118 | -0.817 | 1.429 | -0.572 | 0.575 |
|  | *Forest: PLA* | -6.198 | 2.845 | -2.179 | **0.044** | -14.556 | 14.759 | -0.986 | 0.338 | -4.144 | 1.375 | -3.015 | **0.008** |
|  | *Sand* | -0.009 | 0.005 | -1.585 | 0.131 | -0.025 | 0.028 | -0.877 | 0.393 | -0.06 | 0.026 | -2.282 | **0.036** |
|  | *Slope* | 0.142 | 0.224 | 0.634 | 0.535 | -1.918 | 1.161 | -1.651 | 0.117 | 0.193 | 0.108 | 1.784 | 0.092 |
|  | *Elevation* | -0.067 | 0.042 | -1.571 | 0.135 | -0.254 | 0.22 | -1.154 | 0.264 | -0.035 | 0.021 | -1.718 | 0.104 |
| Trees | *Intercept* | 89.397 | 11.804 | 7.574 | **0** | 135.787 | 61.118 | 2.222 | **0.04** | 34.392 | 16.259 | 2.115 | **0.049** |
|  | *Forest: BN* | -11.899 | 3.783 | -3.146 | **0.006** | 15.411 | 19.586 | 0.787 | 0.442 | -9.558 | 5.21 | -1.834 | 0.084 |
|  | *Forest: RIL* | -15.66 | 4.675 | -3.35 | **0.004** | 0.299 | 24.204 | 0.012 | 0.99 | -8.477 | 6.439 | -1.317 | 0.205 |
|  | *Forest: PLA* | -70.154 | 4.498 | -15.598 | **0** | 231.092 | 23.288 | 9.923 | **0** | -28.835 | 6.195 | -4.654 | **0** |
|  | *Sand* | -0.002 | 0.009 | -0.251 | 0.805 | -0.02 | 0.045 | -0.454 | 0.656 | 0.073 | 0.119 | 0.61 | 0.55 |
|  | *Slope* | 0.202 | 0.354 | 0.571 | 0.576 | 1.697 | 1.833 | 0.926 | 0.367 | 0.022 | 0.487 | 0.046 | 0.964 |
|  | *Elevation* | -0.113 | 0.067 | -1.685 | 0.11 | 0.467 | 0.348 | 1.342 | 0.197 | -0.055 | 0.092 | -0.594 | 0.56 |
